# M2 tumour-associated macrophages contribute to tumour progression via legumain remodelling the extracellular matrix in diffuse large B cell lymphoma

**DOI:** 10.1038/srep30347

**Published:** 2016-07-28

**Authors:** Long Shen, Honghao Li, Yuzhi Shi, Dekun Wang, Junbo Gong, Jing Xun, Sifan Zhou, Rong Xiang, Xiaoyue Tan

**Affiliations:** 1 Department of Pathology Medical School of Nankai University, 94 Weijin Road, Tianjin 300071, China; 2 Department of Immunology, Medical School of Nankai University, 94 Weijin Road, Tianjin 300071, China; 3 Tianjin Key Laboratory of Modern Drug Delivery and High Efficiency in Tianjin University, 92 Weijin Road, Tianjin 300072, China

## Abstract

Effects of M2 tumour-associated macrophages on the pathogenesis of diffuse large B cell lymphoma (DLBCL) are still controversial. Our data showed that the number of CD163-positive M2 macrophages correlated negatively with DLBCL prognosis. Macrophage depletion by clodronate liposomes significantly suppressed tumour growth in a xenograft mouse model of DLBCL using OCI-Ly3 cells. Moreover, M2 polarization of macrophages induced legumain expression in U937 cells. Exogenous legumain promoted degradation of fibronectin and collagen I, which was abolished by administration of a legumain inhibitor RR-11a. Overexpression of legumain in Raw 264.7 cells also induced tube formation of endothelial cells in matrigel. In the xenograft mouse model of DLBCL, decreased fibronectin and collagen I, as well as increased legumain expression and angiogenesis were found at the late stage tumours compared with early stage tumours. Co-localization of legumain and fibronectin was observed in the extracellular matrix of tumour tissues. Administration of the legumain inhibitor to the xenograft DLBCL model suppressed tumour growth, angiogenesis and collagen deposition compared with the control. Taken together, our results suggest that M2 tumour-associated macrophages affect degradation of the extracellular matrix and angiogenesis via overexpression of legumain, and therefore play an active role in the progression of DLBCL.

Diffuse large B cell lymphoma (DLBCL) is the most frequent Non-Hodgkin’s lymphoma, accounting for 30–40% of newly diagnosed lymphomas^1^. Although the current standard therapy for DLBCL cures the majority of patients, it is still a fatal malignancy that requires new targets for additional therapeutic options. Studies on the gene expression profiles of DLBCL biopsy specimens have revealed increased infiltration of macrophages into DLBCL stroma^2^,^3^. Diverse polarized subtypes of macrophages occur in the tumour microenvironment, and there is functional heterogeneity between M1 and M2 macrophages. However, the mechanism underlying the effect of M2 macrophages on the pathogenesis of DLBCL remains unclear^4^,^5^,^6^.

Several clinical data suggest that a high density of tumour-associated macrophages (TAMs), especially M2 TAMs can predict adverse outcomes in many kinds of cancers^7^,^8^. In invasive pancreatic cancer, CD163-positive M2 TAMs, but not CD68-positive macrophages, are associated with a poor prognosis^9^. Similarly, a higher ratio of CD163- to CD68-positive macrophages in angioimmunoblastic T-cell Lymphoma correlates significantly with poor overall survival^10^. Paradoxically, Hasselblom *et al*. suggested that CD68-positive TAMs have no prognostic value in DLBCL^11^. Further study on the correlation between the clinical stage as well as other prognostic indexes and M2 TAMs is urgently needed.

Legumain is an asparaginyl endopeptidase that is classified as a member of the C13 family of cysteine proteases^12^. Accumulating data have identified legumain expression in a variety of tumour types, suggesting a role in tumour progression^13^. Furthermore, studies have verified the effect of legumain on modulation of tumour- and stroma-derived components of the cancer degradome^14^,^15^. A recent study on the crystal structure revealed multi-branched and context-dependent activation processes of legumain, suggesting broad roles in addition to its primary role as an endolysosomal cysteine proteinase^16^. TAMs have been found to express abundant amounts of legumain. A vaccine or inhibitor against legumain significantly eliminates M2 TAMs and consequently inhibits tumour growth and metastases in murine tumour models^17^,^18^.

Here, we provide evidence that a high percentage of M2 TAMs predicts poor outcomes in DLBCL patients. M2 TAMs in the lymphoma microenvironment overexpressed and secreted legumain. In addition, legumain was involved in angiogenesis and tumour progression via extracellular matrix (ECM) remodelling. Therefore, we have identified a novel therapeutic target as well as a diagnostic index for the treatment of DLBCL.

## Results

### A high density of CD163-positive M2 TAMs is a poor prognostic factor in DLBCL

**Figure 1:**
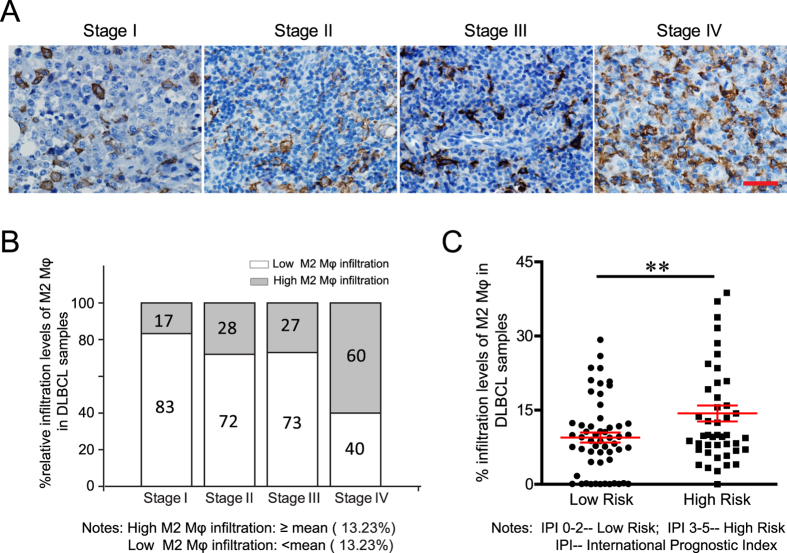
High density of CD163-positive (M2) TAMs is a poor prognostic factor in DLBCL. Immunohistological staining was performed on tissue samples from DLBCL patients at various disease stages. (**A**) Representative images of immunohistological staining for CD163. Scale bar = 100 μm. (**B**) Statistical analysis showed the distribution of the percentage of the CD163-positive staining area in samples at various disease stages. (**C**) Statistical analysis showed the correlation of the IPI and percentage of the CD163-positive staining area. **P < 0.01.

Whether a higher ratio of CD163- or CD68-positive macrophages can serve as a predictive index for a poorer prognosis of DLBCL is still controversial. Here, we collected clinical data from 139 DLBCL patients with various stages of disease and calculated their international prognostic index (IPI). In the tumour samples, immunohistochemical staining of CD163 was performed to visualize the CD163-positive macrophages. Our data showed various ratios of CD163-positive macrophages in DLBCL tumour tissues. The mean ratio of CD163-positive M2 macrophages was 13.23% (Fig. 1A, range: 7–50.5%). Compared with stage I, II and III patients, more stage IV patients showed a high percentage of CD163-positive M2 macrophage infiltration (Fig. 1B, 60% vs 27%, 28%, and 17%). Moreover, the percentage of M2 TAMs in low risk groups was significantly lower than that in the high risk group as evaluated by the IPI (Fig. 1C, 9.459 ± 1.014% vs 14.34 ± 1.634, p = 0.0099). These findings suggest that the ratio of CD163-positive M2 TAMs correlates positively with the disease progression and prognosis of DLBCL.

### M2 TAMs overexpress legumain together with changes in ECM deposition and angiogenesis in the OCI-Ly3 xenograft mouse model of DLBCL

**Figure 2:**
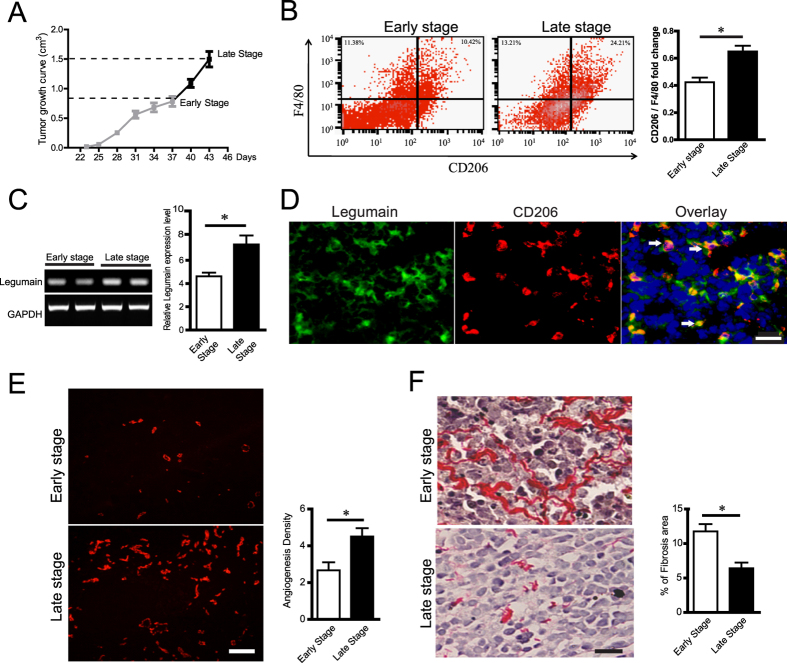
Legumain overexpression in M2 macrophages is accompanied by angiogenesis and less accumulation of ECM components in the OCI-Ly3 xenograft mouse model of DLBCL. The OCI-Ly3 xenograft mouse model of DLBCL was established and tumours were collected when the tumour size had reached 0.8 or 1.5 mm^3^ (n = 5). Changes in the tumour size, ECM deposition, angiogenesis, and legumain expression were measured. (**A**) Tumour growth curve of the OCI-Ly3 xenograft mouse model of DLBCL. (**B**) Flow cytometry analysis of CD206 vs F4/80 expression in the tumour tissues collected at different stages in the DLBCL animal model. (**C**) Representative images and statistical results of RT-PCR analyses of legumain mRNA expression at different stages. (**D**) Representative images of immunofluorescence staining for legumain (green) and CD206 (red). The arrows indicate co-localization of CD206 and legumain. Scale bar = 50 μm. (**E**,**F**) Statistical analysis and representative images of immunofluorescence staining for CD31 and Sirius red staining in tumour tissues samples at different stages. Scale bar = 100 μm. Scale bar = 50 μm. Data are shown as the mean ± s.e.m, *P < 0.05 and ***P < 0.001.

To explore the mechanism underlying the effect of M2 TAMs on the pathogenesis of DLBCL, especially the role of legumain, we established an OCI-Ly3 xenograft mouse model of DLBCL. The tumour growth curve is shown in Fig. 2A. Animals were sacrificed and primary tumour samples were collected when the tumour sizes reached 800 mm^3^ or 1500 mm^3^ (n = 5, separately). Flow cytometry analysis suggested that the ratio of CD206-positive M2 TAMs increased with tumour progression (Fig. 2B). Furthermore, we determined the expression of legumain mRNA in the homogenates of tumour tissues. Legumain mRNA levels in the early stage group were significantly lower than those in the late stage group (Fig. 2C). Immunohistochemical staining of legumain and CD206 showed co-localization of legumain and CD206, suggesting legumain expression in M2 TAMs (Fig. 2D). In addition, we identified a change in the ECM contents with tumour progression in this model. As shown in Fig. 2E, immunofluorescence staining of CD31 suggested an increase in blood vessel density with tumour progression. Sirius red staining was performed to identify the collagen content in tumour tissues. Figure 2F shows that the percentage of the collagen area was higher at the early stage than at the late stage. These data suggested remodelling of the ECM, including angiogenesis, and less deposition of collagen with the progression of DLBCL. Furthermore, the increased number of M2 TAMs overexpressed the protease legumain.

### Legumain catabolizes ECM components including fibronectin and collagen I

**Figure 3:**
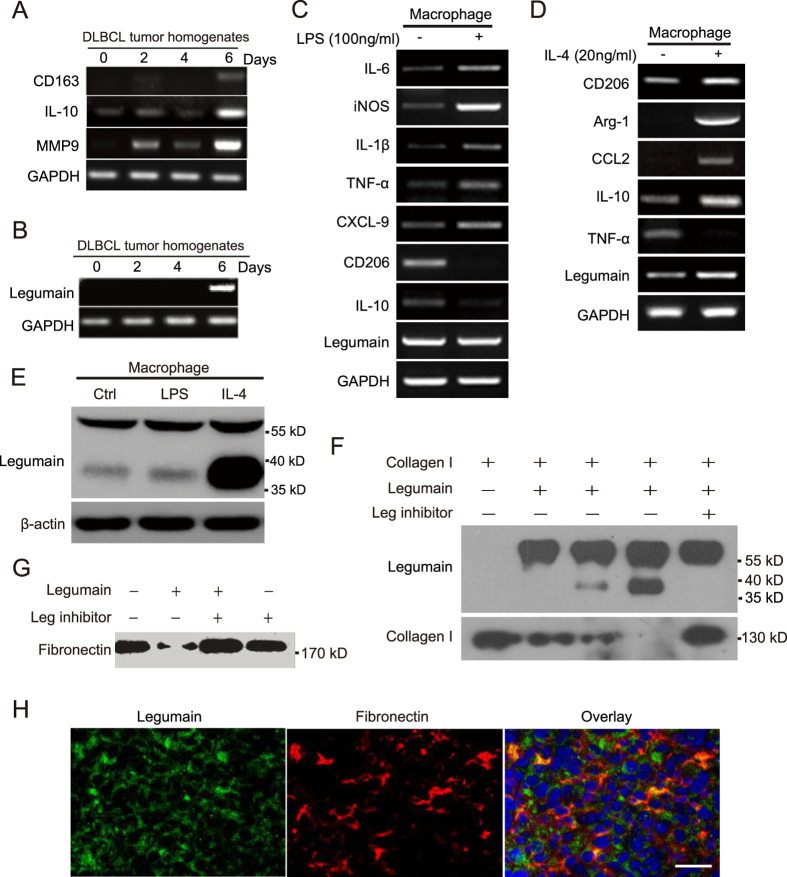
Tumour tissue homogenates induce M2 polarization and legumain expression in macrophages, and legumain degrades fibronectin and collagen I *in vitro*. U937 cells were treated for various periods with tumour homogenates isolated from DLBCL animal models. RT-PCR analysis of CD163, IL-10, MMP9 mRNA expression (**A**) and legumain mRNA expression (**B**) in U937 cells. (**C**) RT-PCR analysis of mRNA expression changes of IL-6, iNOS, IL-1β, TNF-α, CXCL-9, CD206, IL-10 and legumain in mouse primary macrophages after treating with LPS (100 ng/ml) for 24 h. (**D**) RT-PCR analysis of mRNA expression changes of CD206, Arg-1, CCL2, IL-10, TNF-α and legumain in mouse primary macrophages after treating with IL-4 (20 ng/ml) for 96 h. (**E**) Western blotting detect the expression of legumain in mouse primary macrophages in different polarization conditions. (**F**,**G**) Fibronectin and collagen I were treated with legumain for 4 h with or without the legumain inhibitor RR-11a. Western blotting detects the amounts of collagen I, fibronectin and legumain. (**H**) Immunofluorescence staining of legumain (green) and fibronectin (red) in tumour tissue samples from the OCI-Ly3 xenograft mouse model. Scale Bar = 50 μm.

To further identify the role of legumain in ECM remodelling, we tested the effect of legumain on the major components of the ECM, fibronectin and collagen I, *in vitro*. Cultured human U937 monocytic cells were treated for various periods with homogenates of tumour samples from the OCI-Ly3 xenograft mouse model of DLBCL. Our results showed that 6 days of treatment with tumour homogenates induced M2 macrophage makers, CD163, IL-10 and MMP9 expression, as well as the expression of legumain in U937 cells (Fig. 3A,B). Next, we treated isolated primary mouse peritoneal macrophages with LPS or IL-4 to stimulate M1 or M2 polarization, respectively. With the M1 polarization induced by LPS (increased expression of IL-6, iNOS, IL-1β, TNF-α and CXCL-9; decreased expression of CD206 and IL-10), no change on the mRNA level of legumain was detected compared with control (Fig. 3C). However, M2 polarized macrophages induced by IL-4 (increased expression of CD206, Arg-1, CCL2 and IL-10; decreased expression of TNF-α) express more legumain in the mRNA level (Fig. 3D). Also, data from the western blot assay suggested that M2 polarization condition of macrophages induced expression of legumain (Fig. 3E). Furthermore, treatment with legumain catabolized collagen I and fibronectin. These effects were rescued by administration of a synthetic legumain inhibitor, RR-11a^19^,^20^ (Fig. 3F,G). *In vivo*, results of immunofluorescence staining showed co-localization of legumain and fibronectin in the xenografted tumour tissues, suggesting extracellular localisation of legumain in the tumour microenvironment (Fig. 3H). Thus, our data demonstrated that legumain was overexpressed by M2 polarized macrophages in the tumour microenvironment and the capacity of legumain to catabolize ECM components outside of the cell membrane.

### Overexpression of legumain in macrophages stimulates angiogenesis *in vitro*

**Figure 4:**
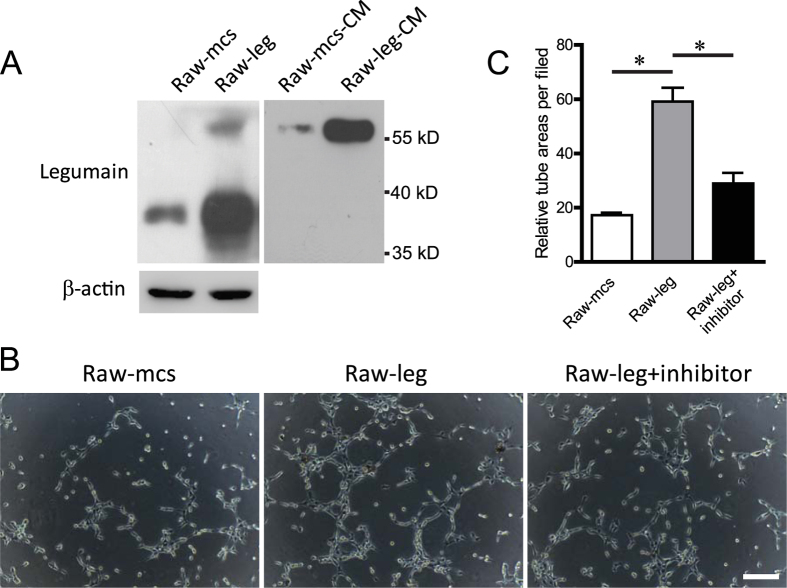
Ectopic overexpression of legumain in RAW 264.7 macrophages stimulates angiogenesis *in vitro*. A stable legumain-overexpressing RAW 264.7 cell line was established. Cell lysates and conditioned medium were collected. (**A**) Western blotting of legumain in cell lysates and conditioned medium. (**B**,**C**) Tube formation assays using HUVECs were performed with addition of conditioned medium from legumain-overexpressing RAW 264.7 cells or the control with or without the legumain inhibitor RR-11a. Representative images and statistical results are shown. Scale Bar = 100 μm. Data are shown as the mean ± s.e.m, *P < 0.05.

Neovascularisation is a critical process during tumour progression. To elucidate the role of legumain overexpressed by M2 TAMs in the angiogenesis of DLBCL, we established a stable legumain-overexpressing RAW 264.7 macrophage cell line. Overexpressed legumain was found in both the cell lysates and conditioned medium of legumain-overexpressing RAW 264.7 cells (Fig. 4A). Next, we examined the effect of legumain overexpressed by macrophages on angiogenesis using endothelial cell tube formation assays. The results showed that pretreatment with conditioned medium from legumain-overexpressing RAW 264.7 cells stimulated the tube formation of HUVECs, and the effect was abolished by administration of the legumain inhibitor RR-11a (Fig. 4B,C).

### Inhibition of legumain suppresses tumour progression in the mouse model of DLBCL

**Figure 5:**
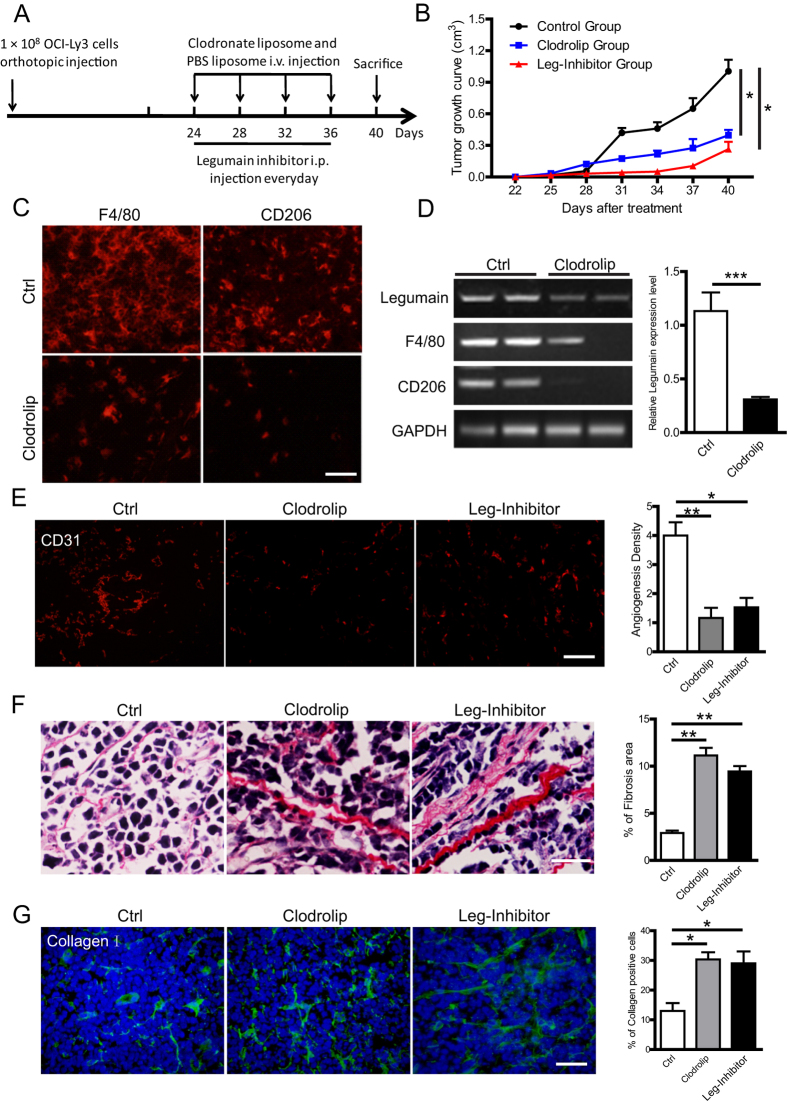
Depletion of macrophages or administration of the legumain inhibitor suppresses tumour progression in the OCI-Ly3 xenograft mouse model of DLBCL. The OCI-Ly3 xenograft mouse model of DLBCL was treated with clodrolip (25 mg/kg), the legumain inhibitor (0.2 mg/kg), or the vehicle only (control). (**A**) Schematic of the experimental design. (**B**) Tumour growth curves. (**C**) Immunofluorescence staining of F4/80 and CD 206 in clodrolip and control groups. Scale Bar = 50 μm. (**D**) RT-PCR analysis of F4/80 and CD206 mRNA expression. The right panel shows the statistical results. (**E**) Immunohistological staining of CD31. The left panel shows representative images and the right panel is the statistical result. Scale Bar = 100 μm. (**F**,**G**) Representative images and statistical bar graph of sirus red staining and immunohistological staining of collagen I. Scale Bar = 100 μm. Data are shown as the mean ± s.e.m. *P < 0.05, **P < 0.01 and ***P < 0.001.

To further explore the effect of legumain expressed by M2 TAMs on the tumour pathogenesis of DLBCL, we established the OCI-Ly3 xenograft mouse model of DLBCL and divided the animals into three groups (n = 5). Clodrolip (25 mg/kg), RR-11a (0.2 mg/kg), or the vehicle only (control liposome) was used to treat mice. As shown in Fig. 5B, there existed significant suppression of tumour growth in groups with macrophage depletion (clodrolip) or legumain inhibition compared with the control group. Immunofluorescence staining identified low infiltration of F4/80-and CD206-positive M2 macrophages in the clodrolip group (Fig. 5C). Similarly, RT-PCR also showed decreased mRNA levels of F4/80 and CD206, as well as suppression of legumain (Fig. 5D). CD31 staining revealed less angiogenesis in clodrolip and RR-11a groups compared with the control (Fig. 5E). Sirus red and immunofluorescence staining of collagen I showed more deposition of collagen in the tumour tissue of clodrolip and RR-11a groups compared with the control (Fig. 5F,G). Taken together, these data suggested inhibitory effects of both macrophage depletion and suppression of legumain on angiogenesis and a protective role against deposition of collagen in the ECM.

## Discussion

As the largest cell population in the tumour microenvironment, the effects of macrophages on tumour pathogenesis have been widely studied in various tumour models. Several lines of evidence support that a high ratio of M2 polarized macrophages (M2 TAMs) leads to a poor outcome of DLBCL. Marchesi and colleagues suggested high M2 TAM levels at diagnosis as an unfavourable prognostic factor in patients with DLBCL^20^. In this study, we evaluated correlations between CD163-positive M2 TAMs and other prognostic indexes of DLBCL. Our data show that different ratio of CD163-positive M2 TAMs exist in tumour tissue samples of DLBCL patients with various disease stages. Moreover, the number of M2 TAMs correlated positively with the IPI. Although tight correlation has been established in Hodgkin’s lymphoma and several other types of haematological tumours, role of TAMs in DLBCL is still controversial till now^21^,^22^. Our data support the density of CD163-positive M2 TAMs as a potential prognostic index in DLBCL. Obviously, further research including a well-defined cut off value, as well as enrolling more patients is needed before its application in clinical practice.

ECM remodelling, including ECM cleavage, modulating angiogenesis and immune responses provides essential signals for tumour progression^23^. Cleavage of ECM components is performed by various families of proteases^24^. Recent studies have revealed that, in addition to matrix metalloproteinases, cathepsins also target many ECM proteins. Our previous study revealed overexpression of a member of the cysteine proteases, legumain, in the tumour microenvironment^25^. Interestingly, not only tumour cells undergoing hypoxia, but also TAMs were the sources of legumain. In this study, co-localization of the M2 TAM marker CD206 and legumain supported that legumain is overexpressed in the tumour microenvironment of DLBCL. Moreover, the expression level of legumain increased with expansion of the tumour, cleavage of the ECM and angiogenesis. *In vitro,* experiments showed that conditioned medium from M2 TAMs induced cleavage of ECM and formation of vessel tubes. Moreover, administration of the legumain inhibitor indicated that these effects were mediated by legumain. At this time, it is still hard to distinguish the major source of legumain in the tumour microenvironment since both tumour cells themselves and tumour associated macrophage have been proven to overexpress this protein. Lin *et al*. revealed that stress stimuli induced TRAF6 ubiquitination of pro-legumain by K63 polyubiquitin thus enhanced both the intracellular stability and secretion of legumain in the tumour cells^26^. Notably, legumain is synthesized as inactive zymogen in the tumour cells while mainly exists in the active form in macrophages, therefore imply a context-dependent action pattern of legumain in different cellular types. Additional investigation is obviously needed to elucidate the exact role and underlying mechanism of action for legumain.

Studies on cathepsins show that they either degrade ECM proteins via being secreted into the extracellular area or internalize ECM components such as collagen through endocytosis and degrade them in lysosomes^27^. As one of the conserved cathepsins, the functional pattern of legumain during the ECM remodelling is still unknown. It is well accepted that legumain is activated in lysosomes intracellularly^28^. However, our study revealed co-staining of legumain and fibronectin in the tumour microenvironment. Moreover, administration of legumain induced cleavage of fibronectin and collagen I. These data support that legumain performs its function outside of the cell membrane. It seems like conflicted with the maintenance of proteolytic activity and stability of legumain. Interestingly, a recent study on the crystal structure revealed different activation routes for legumain, which explained the pH stability of legumain and its extracellular activities to some extent^16^. In addition, recent studies also suggest that exosomes, 30–100 nm-sized membrane vesicles that originate from multivesicular bodies inside of cells, also mediate the extracellular proteolysis by proteases^29^,^30^. Hakulinen reported that matrix metalloproteinase-14 is secreted from exosomes into the extracellular space and degrades type I collagen^31^. Whether legumain is also transported via exosomes, thus reaching the extracellular space, is worthy of further study. Besides degrading ECM components, our data showed that legumain promoted tube formation by endothelial cells on the surface of matrigel *in vitro*. It has been reported that pericellular proteases contribute to cleavage of the ECM and the release of angiogenic factors such as vascular endothelial growth factor. Meanwhile, the degradation products might also directly promote angiogenesis.

Considering the critical role of TAMs, especial M2 TAMs in the progression of tumours, diverse strategies aimed to delete M2 TAMs have been designed and achieved encouraging results on limiting tumour progression^21^. Several studies have reported the effects of cathepsin inhibitors on tumour characteristics. Blocking cathepsin activity with multiple small molecule inhibitors results in suppression of the bone metastasis of breast cancer^32^. In this study, we deleted TAMs using clodronate liposomes or blocked legumain activity using a legumain inhibitor in a xenograft mouse model of DLBCL. Both methods delayed xenograft progression significantly with more ECM deposition and less angiogenesis.

In this study, we identified the M2 macrophage marker CD163 as a potential prognostic index for DLBCL. Moreover, legumain secreted by M2 TAMs contributes to cleavage of the ECM as well as angiogenesis in tumour microenvironments. Administration of a legumain inhibitor or deletion of M2 TAMs limits tumour expansion, ECM cleavage and angiogenesis. Therefore, our study suggests a novel potential method to optimize the treatment of DLBCL.

## Material and Methods

### Cell culture and cell lines

Human DLBCL cell line OCI-Ly3 was kindly provided by Jun Chen (Lung Cancer Research Centre of General Hospital, Tianjin, China) and cultured in RPMI 1640 supplemented with 20% fetal bovine serum (FBS). The human monocytic cell line U937, human umbilical vein endothelial cells (HUVECs; a primary endothelial cell line) and the mouse monocytic cell line Raw 264.7 were kind gifts from Dr. Ralph Reisfeld (Scripps Research Institute, La Jolla, CA). U937 and Raw 264.7 cells were cultured in RPIM 1640 supplemented with 10% FBS. HUVECs were cultured in EGM-2 endothelial cell medium (LONZA, Basel, CH). Lentivirus production was performed as described previously^33^. To establish a stable legumain-overexpressing cell line, Raw 264.7 cells were transfected with the pLV-EF1α-legumain-IRES-Bsd plasmid. A control cell line containing the empty vector was established by the same methods described previously^33^.

### Animal models of tumours

Six- to eight-week-old male NOD/SCID mice were purchased from Beijing HFK Bio-Technology (Peking, China) and housed at the Animal Centre of Collagen of Life Science, Nankai University. All animal experiments were approved by the Institutional Animal Care and Use Committee at Nankai Univeristy, and were performed in accordance with relevant guidelines and regulations. To establish the animal model, 100 μl of OCI-Ly3 cells (1 × 10^8^ cells/ml) was subcutaneously injected at the forth rib of mice. For tumour staging, mice were divided randomly into two groups (n = 5) and sacrificed when the lymphoma volume reached 800 mm^3^ (early stage) or 1,500 mm^3^ (late stage). To analyze the effect of M2 TAMs and legumain on tumour progression, mice were divided randomly into three groups (n = 5) and intravenously injected with PBS (control) or clodronate liposomes (25 mg/kg) every 4 days (clodronate liposomes and PBS liposomes were provided by Nicovan Rooijen, Amsterdam, Netherlands); or intraperitoneally injected with legumain inhibitor RR-11a (0.2 mg/kg) every day for 12 days. Tumour growth curves were recorded and the mice were sacrificed at the end of the experiments. Primary tumours were collected for tissue sectioning and homogenate preparation.

### Tissue processing and Sirius red staining

Tumour tissues were fixed with 3.7% paraformaldehyde, embedded in paraffin, and sectioned. For Sirius red staining, nuclei were stained with Weigert’s haematoxylin for 15 2 min, followed by washing under running water for 10 min. Collagen was stained with picrosirius red (Abcam, Cambridge, MA) for 1 h, washed with acidified water, dehydrated, and mounted in a resinous medium. Images were captured by a BX53 research microscope (Olympus, Tokyo, Japan).

### Isolation and polarization of mouse peritoneal macrophages

To isolate murine peritoneal macrophages, inject 1 ml of 3% Brewer thioglycollate medium into the peritoneal cavity of each mouse. 4 days later, the mice were injected with 5 ml of ice cold PBS (with 3% FBS) into the peritoneal cavity. Then the peritoneal fluid was withdrawn slowly and centrifuged at 1500 rpm for 8 min, the cells were resuspended in RPMI 1640 supplemented with 10% FBS. To stimulate M1/M2 polarization in macrophages, 100 ng/ml lipopolysaccharide (LPS; Sigma-Aldrich, St Louis, MO) or 20 ng/ml mouse interleukin 4 (IL-4; R&D, Minneapolis, MN) were added to the macrophages and incubated for 24 h or 96 h at 37 °C.

### Flow cytometry

OCI-Ly3 xenograft tumour tissues were collected, digested, and filtered to prepare mono-cellular suspensions. To quantify the ratio of CD206-positive TAMs in primary tumour cells, cells were washed twice with PBS containing 1% FBS, dispersed cells were incubated with PE/Cy5 anti-mouse F4/80 (Biolegend, San Diego, CA) and FITC anti-mouse CD206 (Biolegend) antibody for 15 min on ice. The resulting suspensions were immediately analysed using a digital BD Caliber flow cytometer (BD Biosciences, San Jose, CA).

### RNA extraction and polymerase chain reaction (PCR) analysis

OCI-Ly3 xenograft tumour tissues were ground and filtered to prepare tumour homogenates. U937 monocytic cells were treated with the tumour homogenates. RNAs from lymphoma tissues. U937 cells and primary macrophages were isolated using Trizol Reagent (Ambion, Carlsbad, CA) and reverse transcribed to cDNA with a reverse transcription system (TransGen Biotech, Peking, China). Then, we conducted semi-quantitative PCR by agarose gel electrophoresis. The primers used were as follows (5′–3′, sense & anti-sense): h-legumain, ggcacagatgtctatcagggag and ctcatcatagtaacaggcgtaggac; h-CD163, cgagttaacgccagtaagg and gaacatgtcacgccagc; h-IL-10, ggttgccaagccttgtctga and agggagttcacatgcgcct; h-MMP9, atgcgtggagagtcgaaatc and tacacgcgagtgaaggtgag; h-GAPDH, gcaccgtcaaggctgagaac and tggtgaagacgccagtgga; m-IL-1β, atccagcttcaaatctcgc and atctcggagcctgtagtgc; m-iNOS, gaaacgcttcacttccaatg and aatccacaactcgctccaa; m-IL-6, ttgccttcttgggactgat and ttgccattgcacaactctt; m-CXCL-9, tcatcttcctggagcagtg and ttcttcacatttgccgagt; m-Arg-1, cagtctggcagttggaagc and ttggcagatatgcagggag; m-IL-10, caacatactgctaaccgactc and catggccttgtagacacct; m-legumain, cgccgtgctgagaggtgacg and gactccttggggttggccgc; m-F4/80, cgtgctggagcaagcgacca and tctccccggtcacagtgcca; m-CD206, acgcagtggttggcagtggg and ttgccaggtccccaccctcc; m-TNF-α, cctcttctcattcctgcttg and cacttggtggtttgctacg; m-CCL2, cctgctgttcacagttgcc and tggacccattccttcttgg; m-GAPDH, tgcccagaacatcatccct and gaagtcgcaggagacaacc.

### Immunohistochemical and immunofluorescence analyses

The detailed procedures of immunohistochemical and immunofluorescence analyses have been described previously^34^. Protein expression was detected in OCI-Ly3 xenograft tissue sections with antibodies against F4/80 (Santa Cruz Biotechnology, Santa Cruz, CA), CD206 (eBioscience, San Diego, CA), CD31 (BD Biosciences), collagen I (Abcam), and legumain (Santa Cruz Biotechnology). Specimens from DLBCL patients were fixed and immunohistochemically stained for CD163 (BD Biosciences). Images were captured by an IX51 research microscope. (Olympus)

### Tube formation assay

HUVECs were used in the tube formation assay. Briefly, matrigel was coated onto 48-wells plates and then incubated at 37 °C with 5% CO_2_ for 30 min to solidify the matrigel. Once the matrigel had set, 200 μl Raw 264.7 cell-derived conditioned medium was mixed with 50 μl resuspended HUVEC cells (approximately 2 × 10^4^ cells) and then added to the plates. After 6 h, the wells were washed with PBS and the tube network was imaged using the IX51 research microscope.

### ECM degradation and western blot analysis

Fibronectin and collagen I were coated to the bottom of 96-well plates and treated with or without legumain for 2 h. Then, protein samples were collected and subjected to western blotting with antibodies against fibronectin (Santa Cruz Biotechnology) and collagen I (Abcam). To detect proteins extracted from cells, equivalent amounts of total protein from each lysate were loaded for western blotting to analyze the target protein level with the indicated antibody including anti-legumain and anti-β-actin (Santa Cruz Biotechnology) antibodies. An ECL chemiluminescence kit (Millipore, Billerica, MA) was used to detect the proteins.

### Statistical analysis

GraphPad Software (La Jolla, CA) was used for data analysis. The statistical significance among groups was determined by a t-test and two-tailed analysis of variance. A P-value of less than 0.05 was regarded as statistically significant.

## Additional Information

**How to cite this article**: Shen, L. *et al*. M2 tumour-associated macrophages contribute to tumour progression via legumain remodelling the extracellular matrix in diffuse large B cell lymphoma. *Sci. Rep.* **6**, 30347; doi: 10.1038/srep30347 (2016).
